# Heparanase is a prognostic indicator for postoperative survival in pancreatic carcinoma

**DOI:** 10.1038/sj.bjc.6600504

**Published:** 2002-09-04

**Authors:** J Rohloff, J Zinke, K Schoppmeyer, A Tannapfel, H Witzigmann, J Mössner, C Wittekind, K Caca

## Abstract

*British Journal of Cancer* (2002) **87**, 689–689. doi:10.1038/sj.bjc.6600504 www.bjcancer.com

© 2002 Cancer Research UK

**Correction to:** *British Journal of Cancer* (2002) **86**, 1270. doi:10.1038/sj/bjc/6600232

## 

The authors would like to thank InSight Ltd., Rehovot, Israel for providing them with the anti-heparanase antibody and heparanase cDNA used in the study.

The mentioned reagents are proprietary of InSight.

